# Principles of Glomerular Organization in the Human Olfactory Bulb – Implications for Odor Processing

**DOI:** 10.1371/journal.pone.0002640

**Published:** 2008-07-09

**Authors:** Alison Maresh, Diego Rodriguez Gil, Mary C. Whitman, Charles A. Greer

**Affiliations:** 1 Department of Neurobiology, Yale University School of Medicine, New Haven, Connecticut, United States of America; 2 Department of Neurosurgery, Yale University School of Medicine, New Haven, Connecticut, United States of America; Emory University, United States of America

## Abstract

Olfactory sensory neurons (OSN) in mice express only 1 of a possible 1,100 odor receptors (OR) and axons from OSNs expressing the same odor receptor converge into ∼2 of the 1,800 glomeruli in each olfactory bulb (OB) in mice; this yields a convergence ratio that approximates 2∶1, 2 glomeruli/OR. Because humans express only 350 intact ORs, we examined human OBs to determine if the glomerular convergence ratio of 2∶1 established in mice was applicable to humans. Unexpectedly, the average number of human OB glomeruli is >5,500 yielding a convergence ratio of ∼16∶1. The data suggest that the initial coding of odor information in the human OB may differ from the models developed for rodents and that recruitment of additional glomeruli for subpopulations of ORs may contribute to more robust odor representation.

## Introduction

Central odor processing begins in olfactory bulb (OB) glomeruli where olfactory sensory neuron (OSN) axons converge and synapse onto projection neurons, mitral and tufted cells, and populations of interneurons, peri/juxtaglomerular cells. In rodents, each OSN expresses only 1 of ∼1100 odor receptors (OR) [1], [2], [3]. Moreover, each OSN projects one axon to a single glomerulus [4], and all of the OSN axons innervating a glomerulus express the same OR [5]. Thus, the molecular specificity of OSNs established by OR expression is maintained in molecularly homogeneous target glomeruli, creating a stereotyped glomerular map of ORs, with any 1 OR typically represented by two glomeruli [6], [7], [8].

Odor processing by the OB projection neurons, mitral/tufted cells, is regulated by OB interneurons; peri/juxtaglomerular (PG) cells form dendrodendritic synapses with the primary dendritic arbor of projection neurons, and granule cells with the secondary dendrites in the external plexiform layer (EPL) [9]. These largely inhibitory synapses contribute to the modulation of projection neuron output and local lateral inhibition. The ongoing replacement of these interneurons in the adult reflects the highly dynamic nature of the OB synaptic circuits and their capacity for plasticity.

While rodent models of mammalian OB function are important, it is unclear if these general principles of organization extend to the human, particularly in light of the largely anecdotal arguments that olfaction in humans has been reduced in importance over evolution [10]. As a first step toward a better understanding of the cellular and molecular mechanisms of organization in the human OB and processing of odor information, we have begun analyzing the organization of glomeruli in the human OB.

## Results

### Conservation of OB cellular organization

**Figure 1:**
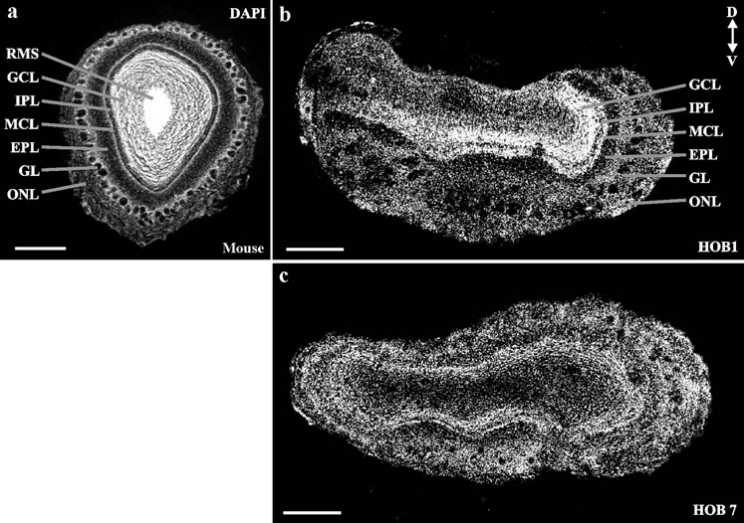
Laminar organization of HOB glomeruli. Laminar organization DAPI stained mouse (a) and human (b and c) OBs. a) Coronal section of the adult mouse OB shows the olfactory nerve layer (ONL), glomerular layer (GL), external plexiform layer (EPL), internal plexiform layer (IPL), granule cell layer (GCL) and rostral migratory stream (RMS). b,c) Coronal HOB sections show equivalent laminae. Scale bars = 500 µm.

In the mouse the laminar organization of the OB is evident (Fig. 1a), with a general consensus regarding the cellular and synaptic organization of each of the layers [9], [11]. A comparable laminar organization is found in the human OB, however, it is less rigorous in the segregation of cell populations and also often lacks the circumferential organization of layers found in rodents and the medial-lateral symmetry of the rodent OB (Fig. 1b,c). As is particularly evident between the OBs shown in Figure 1b and 1c, there is considerable anatomical diversity amongst individual humans.

### Abundance of glomeruli in the human OB

**Figure 2:**
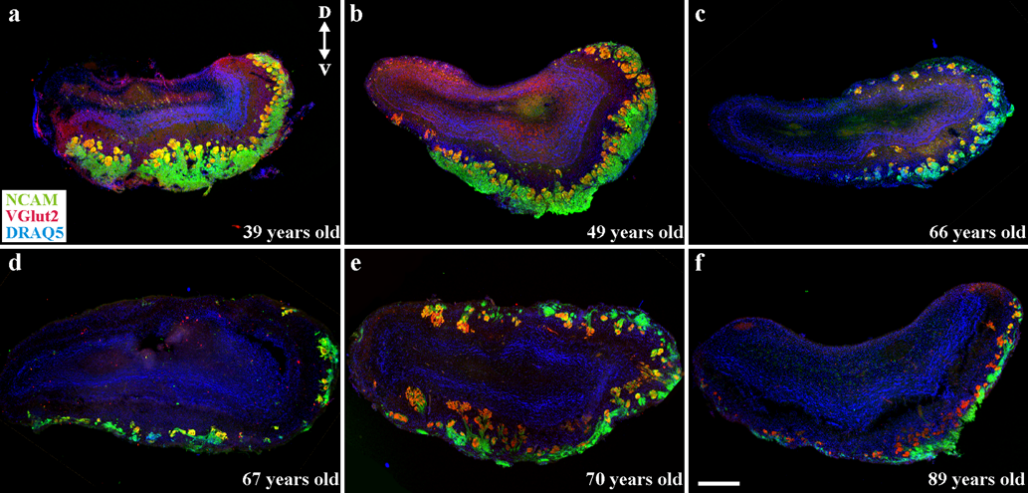
Distribution of glomeruli in the HOB. Triple-labeling for NCAM (green), VGlut2 (red) and DRAQ5 (blue) reveals complex distributions of HOB glomeruli. Scale bar = 250 µm.

**Figure 3:**
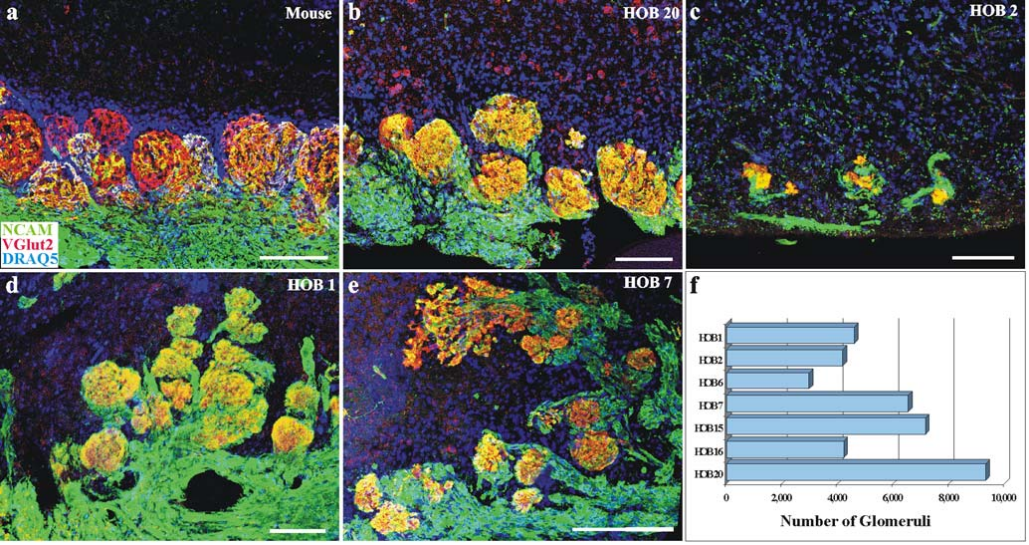
Morphology and variability among HOB glomeruli. Glomeruli shown with NCAM (green), VGlut2 (red) and DRAQ5 (blue). a) Mouse glomeruli are spherical and evenly spaced. b,c,d,e) HOB glomeruli show a broader range of morphology and variability. f) The number of HOB glomeruli. Scale bars = 100 µm.

In rodents, coding of odor signals begins as OSN axons segregate into specific glomeruli in the OB [5], [12], [13]. Do the same principles of OSN axon segregation occur in the human? We first assessed the spatial distribution of glomeruli in OBs from human donors (Fig. 2). In the rodent NCAM identifies the OSN axons while the vesicular glutamate transporter 2 (VGlut2) is specific for the OSN synaptic terminals in rodents [14]; Greer lab unpublished observations. Probes to both NCAM and VGlut2 were successfully adopted and used to identify glomeruli in human OBs (Fig 2). The co-localization (yellow) of VGlut2 and NCAM provided the first definitive marker of OB glomeruli in the human and revealed a level of complexity not anticipated. Consistent with the non-contiguous organization of the OB layers seen in Nissl stains, the nerve and glomerular layers did not extend around the full circumference of the OB. In the youngest samples (i.e. Fig. 2a) the nerve layer appears thick relative to that seen in older samples (i.e. Fig. 2f) but such observations were inconsistent and not statistically significant. In most cases the glomeruli appeared closely apposed to the NCAM^+^ nerve layer of the OB, but there were notable exceptions. For example, as shown in Figure 2e, glomeruli could be found deep in the OB, apparently invading the external plexiform layer and proximal to the mitral or granule cell layers. While many glomeruli were evenly spaced and spherical (Fig. 3b), characteristic of the rodent phenotype (Fig. 3a), glomerular volume was variable (i.e Fig. 3c). Clustering of glomeruli was also noted (Fig. 3d), as were distinct branching phenotypes of invasive glomeruli (Fig. 3e) (cf. Supplementary Fig. S1). In general, OBs from older donors tended to exhibit more atypical or aberrant patterns of glomerular organization than OBs from younger donors. The higher incidence of atypical glomerular patterning among aged donors does suggest that perturbed targeting by OSN axons may contribute to age-associated changes in olfactory function.

We next sought to determine the number of glomeruli in the human OB. In order to ensure that glomeruli were accurately identified, we used the occurrence of double labeling with NCAM and VGlut2 in serial sections through the human OBs as a rigorous and unbiased criteria, corrected with Abercrombie's to further ensure that glomeruli were counted only once. Based on the prevalent rodent model in which each OR is typically represented by 2 glomeruli in each OB, the predicted ratio of glomeruli to ORs is 2∶1. Genomic analyses identified ∼1,200 functional ORs in rats [3], ∼1,100 in mice [3], [15], and ∼350 in humans [15], [16], though the presence of additional OR pseudogenes is prevalent, representing ∼20% of total OR genes in rats and mice [3] and ∼60% in humans 15, 16, 17, 18. Mice have ∼1,800 glomeruli in each OB [19] while rats have ∼2,400 [20], as the model predicts. Based on the 2∶1 model, we hypothesized the human OB would have ∼700 glomeruli, 2 for each of the 350 identified intact ORs in the human genome. However, the number of glomeruli in the human OB was startling (Figure 3f). The human OB had on average 5,568±830 (mean±S.E.M.) glomeruli in each OB with a range of 2,975–9,325 (see Supplementary Table S1 for further quantification). There was no relationship between number of glomeruli and age (p = 0.39), gender (p = 0.66), glomerular diameter (p = 0.71), or total OB volume (p = 0.31) (Supplementary Fig. S2). The large number of glomeruli is striking both in the degree to which it deviates from the predicted model, as well as the degree to which it varies between individual human donors. Notably, the lowest glomeruli count, 2,975, was from HOB 6, a patient with leukemia that had undergone chemotherapy, but no other clinical or lifestyle data available accounted for the variability in glomeruli counts across individuals.

### Similarity of molecular phenotypes and synapses

A hallmark of the rodent olfactory system is the ongoing replacement of OSNs and subpopulations of OB interneurons [21], [22]. To assess neurogenesis in the human OB we tested for GAP-43 expression, a marker of growing immature OSN axons, and NCAM which ubiquitously marks OSN axons in rodents [23], [5]. These markers were found to have a similar distribution in human OBs as in mice (Supplementary Fig. S3). Despite qualitative differences in the lamination and overall organization of the olfactory nerve layer and glomeruli, GAP43/NCAM^+^ axons were readily detected in OB sections from donors aged 39–89 years (i.e. Supplementary Fig. S3a,b). The GAP43^+^ axons distributed around the periphery of glomeruli, consistent with the model in which new OSN axons are integrated from the periphery of existing glomeruli while mature axons are more centrally located (Supplementary Fig. S3c) [23].

As others have recently described, we also found evidence favoring the ongoing genesis and turnover of populations of OB interneurons [24]. Staining for doublecortin, which identifies migrating neuroblasts and both periglomerular and granule cells during the early stages of differentiation, as well as immature OSNs, was robust in the OBs of donors of all ages (Supplementary Fig. S3d). Although there is a continuing controversy regarding the origin of migrating neuroblasts in the human OB [25], our data with doublecortin and GAP43 suggests that adult neurogenesis is indisputable for both OSNs and OB interneurons, and that the human OB is a dynamic structure with a capacity for plasticity throughout life.

Using the axonal marker, NCAM, and the dendritic marker, MAP2, we next evaluated the synaptic organization of glomeruli and demonstrated the presence of axonal and dendritic compartments (Supplementary Fig. S3e). Glomerular compartmentalization was described in the rodent model, and represents the segregation of axodendritic primary afferent synapses and local circuit dendrodendritic synapses [23], [26]. Consistent with the compartmentalization of glomerular synaptic circuits, we found prototypical axodendritic and local circuit dendrodendritic synapses similarly segregated in the human OB. The organization and features of the glomerular synapses in the human OBs (Supplementary Fig. S4). were equivalent to those described in rodents [9]. We also characterized several classes of PG cells using markers for TH and GAD65/67, both key enzymes in pathways of the inhibitory neurotransmitters dopamine and GABA, as well as the calcium binding protein calretinin, all of which have been described in detail in the rodent OB [27], for review. The cellular phenotypes, relative frequencies, and areas of distribution in the human OB are qualitatively similar to those in rodents [28], [29] (Supplementary Fig. S5).

## Discussion

Our data demonstrate an unexpectedly large number of glomeruli in the human OB. The dominant mammalian model suggests ∼2 glomeruli/OR. In the human OB the ratio is ∼16∶1, or greater by a factor of 8. The presence of large and variable numbers of glomeruli may reflect a fundamental difference in human OB organization, perhaps indicating divergence from the rodent model of odor processing. However, despite the large number of glomeruli in the human OB, their intrinsic organization appears comparable to that described in rodents suggesting a general preservation of functional properties. In this context, the large number of glomeruli in the human OB may be a consequence of variations in OR gene evolution as well as the unique experience of odorant exposure.

The large number of glomeruli in the human OB relative to the number of intact ORs in the human strongly suggests that the convergence of axons from the epithelium into the OB in the human differs from that described in the mouse. It remains to be determined if glomeruli in the human OB are molecularly homogeneous for ORs, as occurs in the mouse and rat. Since there are no antibodies specific for individual human ORs, we attempted to address this important question using the limited number of antibodies to raised to mouse ORs that are available. Unfortunately, we were unable to detect any labeled OSN axons with the antibodies to mouse ORs. This may indicate that those ORs are not present in the human, or more simply that mouse OR antibodies lack specificity in human tissue. In either case, resolution of the molecular specificity of the glomeruli in the human OB remains a high priority and one that will need to be addressed before we can fully understand the implications of the large number of glomeruli in the human OB.

The decrease in the size of the OR gene repertoire in humans and the large number of pseudogenes likely reflect a lessening of evolutionary pressure due to a decrease in human dependence upon olfactory cues for survival. This is also suggested by the large degree of sequence variation amongst OR genes in humans. Because even small changes in the amino acid sequence of an OR affects the targeting of axons to glomeruli [12], polymorphisms may increase the number of glomerular representations of a single OR. Moreover, recent evidence suggests that from throughout the genome, as many as 50% of mouse and from 5–20% of human pseudogenes may be transcribed or otherwise functionally active [30]. Consistent with these estimates, Serizawa et al. [31] reported transcription of several documented pseudogenes in the mouse OE. Given the abundance of pseudogenes in the human OR repertoire [15], transcription or other functionality could result in an increase of the heterogeneity of mechanisms regulating OSN axon targeting/coalescence, and thus an increase in the total number of glomeruli.

The continued neurogenesis of the interneuron and OSN cell populations throughout life reflects the dynamic nature of the OB. Such an adaptable system of synaptic plasticity strongly suggests a role for olfactory experience in the organization of the OB. Recent observations [13], [32] suggest that glomerular representation of ORs can be influenced by the odor environment/experience. Given the largely homogeneous nature of odor environments of rodents used in laboratory research versus the more complex odor experiences available to humans, it is tempting to speculate that the individual variation in glomerular number and distribution may be subject, in part, to the individual's personal odor history.

Despite the capacity for ongoing neurogenesis, olfactory function in humans declines steadily after the age of 40 [33], with a 70% prevalence of olfactory dysfunction in the elderly [34]. Little is known about the cellular or molecular mechanisms that may contribute to decreases in olfactory function, and in our analyses of human OBs, there were no statistically significant differences across the ages. This contrasts with Meisami et al. [35] who reported large decreases in the number of glomeruli and mitral cells in the aged; though in those studies the criteria for identifying glomeruli may have been less stringent than those employed here. Meisami et al. [35] processed tissue with a Nissl stain and used the absence of stained somata as the criteria for the presence of a glomerulus. As discussed above, our use of synaptic and axonal markers provides the first definitive measure of glomeruli in the HOB. Despite the absence of significant differences in glomerular number across ages, we did note a qualitative decline in OB lamination and an increase in the incidence of atypical glomeruli among the elderly. These may reflect alterations in the efficacy of OSN axon coalesence/targeting that could contribute to decreases in olfactory function. The etiology of these changes is unclear; however, in a dynamic system undergoing constant neurogenesis and integration of new neurons into complicated synaptic networks, it is plausible that errors would gradually accumulate over the years, and that these would be more pronounced in those with more OSN damage due to chemical exposures or infections.

In conclusion, while odor processing appears to be identical in humans when compared to rodents at the molecular and synaptic level in the OB, there are striking differences in glomerular organization. Further work will clarify the molecularly homogeneous nature of glomeruli, as well as examine the relationship between an individual's olfactory status and the cellular organization of their OB. In addition, a better understanding of human OR genetics as well as the effect of olfactory experience on glomerular organization may better clarify why humans have such large and variable numbers of glomeruli.

## Methods

### Tissue Procurement and Fixation

Post-mortem OBs from autopsy were kindly made available by Dr. Jung Kim from the Department of Pathology, Yale University School of Medicine, New Haven, CT. Information regarding age, gender, and relevant medical history was obtained for all donors (Supplementary Table S2). Exclusions for this part of the study included the presence of symptomatic olfactory dysfunction, neurodegenerative disorders such as Alzheimer's Disease and Parkinson's Disease, and intranasal drug use. The post-mortem interval in these cases was less than 24 hours. After procurement the OBs were fixed in 10% formalin for 7 to 28 days and washed two to three times overnight in fresh phosphate buffered saline (PBS). Procurement of this tissue and relevant donor information passed HIC approval (#0606001589), and is exempt from IRB review.

Live donor OBs were kindly obtained by Dr. Dennis Spencer of the Department of Neurosurgery, Yale University School of Medicine, New Haven, CT, during frontal lobe neurosurgical cases requiring dissection of the lateral olfactory tract (HIC# 12081). OBs were obtained only in those cases in which they would otherwise be sacrificed or discarded during the course of the surgery. These OBs were fixed in 4% paraformaldehyde for 24 to 48 hours, and then washed in PBS overnight.

For qualitative interspecies comparisons, adult CD1 mice (Charles River Laboratories) were anesthetized with sodium pentobarbital (80 mg/kg i.p.; Nembutal; Abbott Laboratories, North Chicago, IL), then decapitated. Perfusions were not performed to more closely replicate the conditions under which the human OBs were prepared. The mouse brains were removed from their skulls, and their OBs were removed and placed in 4% paraformaldehyde overnight, followed by PBS overnight. All procedures undertaken in this study were approved by Yale University's Animal Use and Care Committee and follow NIH guidelines.

After fixation, all human and mouse OBs were cryo-preserved in 30% sucrose in PBS for 12 to 24 hours, then sectioned coronally throughout the length of the entire OB on a sliding-freezing microtome (50 µm). Slices were maintained in rostral-caudal order and stored at −20°C until use.

### Immunohistochemistry

Tissue was removed from −20°C storage and washed in PBS with 0.3% Triton 100-X (PBS-T). For antigen retrieval, OB slices were steamed for 10 minutes in a solution of 0.01 M Sodium Citrate, then immediately washed with PBS-T. Tissue was blocked with 2% BSA in PBS-T for 30 to 60 minutes, then incubated for 48 to 72 hours in primary antibody diluted in BSA-PBS-T at 4°C. We used antibodies against MAP2 (1∶1000, Sigma), GAP43 (1∶1000, Sigma), NCAM (1∶500, Sigma), VGlut2 (1∶4000, Synaptic Systems), DCX (1∶1000, Santa Cruz), Calretinin (1∶400, Chemicon), GAD65/67 (1∶1000 Stressgen), and TH (1∶1000, Chemicon). Tissue was then washed in PBS-T, and incubated in secondary antibody diluted in BSA-PBS-T for 2 hours along with a nuclear marker, DAPI (Sigma) and/or DRAQ5 (Alexis Biochemicals). The sections were then washed in PBS-T, then PBS. In order to eliminate autofluorescence from lipofuscin granules, sections were stained with 1% Sudan Black in 70% Methanol for 5 minutes, then cleared in 70% Ethanol and rinsed in PBS [36]. Sections were mounted with GelMount (Bioveda), and images were taken with a Leica confocal microscope. All staining was done in at least four different human OBs, and presented images are typical unless stated otherwise.

### Quantifying Glomeruli

Glomeruli were defined by colocalization of antibodies against NCAM and VGlut2, and were quantified from every sixth section throughout the length of the human OBs. Overlapping images were taken circumferentially around each section with an Olympus BX51 epifluorescent microscope using the 20× objective. Glomeruli were manually identified on these digitized images, then analyzed using Metamorph software (Molecular Devices, Sunnyvale, CA) to calculate total numbers of glomeruli as well as area and length/width diameters of each glomerulus.

The length of the OB was defined by the distance encompassed by the most rostral and most caudal OB sections that exhibited glomerular staining. The volume was calculated by estimating the shape of the OB to be a cylinder, and the cross sectional area was estimated by averaging the area of 4 slices distributed through the length of the OB. The total counted glomeruli per OB was calculated by first multiplying the total number of counted glomeruli from the sections looked at by the inverse of the fraction of slices counted, usually around 6 as about every 6^th^ slice was selected for counting. Finally, to correct for the glomerular overlap between sections, the Abercrombie extrapolation was used: N = n * (t/(t+H)), where in this case N is the number of glomeruli in the OB, n is the total number of counted glomeruli, t is the width of each section (50 µm) and H is the average glomerular diameter.

Statistical analyses were performed using the Prism package (GraphPad Software Inc., San Diego, CA). To look for relationships between the number of glomeruli and the age of the donors, the size of their glomeruli, or the volume of their OB, a linear regression test was performed. To look for significance between the mean number of glomeruli in male donors versus female donors, as well as between “young” donors (<50 years old) and “elderly” donors (>50 years old), an unpaired t-test was performed. There were no significant differences in the variances in either of these comparisons.

## Supporting Information

Table S1 Olfactory bulb analysis data
(0.05 MB DOC)

Table S2 Olfactory bulb donor information
(0.04 MB DOC)

Figure S1 Additional glomerular phenotypes Additional examples of glomeruli from HOBs labeled with NCAM (green) and VGlut2 (red). Glomeruli were often regularly spherical and regularly distributed (a), though sometimes clustered in groups that make increase the difficulty of distinguishing individual glomeruli and their size and shape (b, c). A further example emphasizes the complexity of glomerular organization and penetration into the deep layers of the HOB (d). Scale bars are 100 µm in a–d.
(5.87 MB TIF)

Figure S2 Relationships between total glomerular and age, gender, glomerular diameter, and OB volume No significant relationships were found between total glomeruli and donor age (p = 0.39) (a). There was a trend towards decreasing numbers of glomeruli with increasing age, however even when dichotomized into two groups of young (age less than 50 years old) and elderly (age greater than 50 years old), there was not a significant difference (p = 0.33). The average number of glomeruli in OBs from the young group was 6,960±2,365 (n = 2), while in the elderly group it was 5,012±785 (n = 5) (b). When grouped by gender, the mean number of glomeruli in OBs from female donors was 6,047±1,643 (n = 3), and from male donors, 5,210±981 (n = 4), which was also not significant (p = 0.66) (c). Finally, there was no correlation between glomerular number and average glomerular size (p = 0.71) (d), or between glomerular number and OB volume (p = 0.31) (e). Linear regressions were performed to look for significance in a, d, e. Unpaired t-tests were performed for the two-group comparisons in b, c. There were no significant differences in variance for either of these comparisons.
(14.23 MB TIF)

Figure S3 Neurogenesis and intrinsic organization of HOB glomeruli Double labeling with GAP43 (red) and NCAM (green) identifies immature OSN axons in the olfactory nerve layer and in the glomeruli of the HOB from both young (a) and older (b) donors. The immature GAP43+ OSN axons first integrate into the periphery of existing glomeruli, a process previously described in rodents (c). Immature OSN axons are also seen with doublecortin in both the nerve layer and glomeruli (d). Migrating neuroblasts, also identified with doublecortin (green), are seen in the human OB (d) as previously described in mice (inset). The presence of subcompartmental organization within glomeruli, axonal compartments as demonstrated by NCAM (green) and dendritic compartments as demonstrated by MAP2 (red) (e), suggests a further parallel with the intrinsic organization of glomeruli in rodents. Abbreviations as in Figure 1. Scale bars: a = 100 µm in a, b; 25 µm in c, e; 50 µm in d; 500 µm in inset of d.
(6.50 MB TIF)

Figure S4 Synaptic morphology in HOB glomeruli (a) In the HOB olfactory nerve terminals (ont) make typical asymmetrical axodendritic synapses with OB neurons. Clusters of spherical vesicles are seen closely apposed to the presynaptic membrane in the electron dense axon terminals. (b) Mitral cell dendrites in the glomeruli make asymmetrical synapses with the intraglomerular dendrites of periglomerular cells. The clusters of vesicles in the mitral cell dendrite a characteristically small. (c) Periglomerular cell dendrites establish symmetrical synapses with mitral cell dendrites. Note the pleomorphic nature of the synaptic vesicles in the periglomerular cell dendrite. Arrows indicate the polarity of the synapses. Abbreviations: ont, olfactory nerve terminal; mc, mitral cell dendrite; pg, periglomerular cell dendrite. Calibration bar shown in (c) = 1 µm.
(7.71 MB TIF)

Figure S5 Molecular phenotypes and distributions of periglomerular cells In the HOB large TH+ (red) cells surround glomeruli, as defined by NCAM (green) and DRAQ5 (blue) (a) At higher magnification processes from the TH+ neurons are seen extending into a glomerulus (b). Calretinin+ (green) cells have smaller cell bodies and are densely distributed around the circumference of HOB glomeruli identified with VGlut2 (red) (c,d). The EPL is dense with GAD65/67+ (green) processes (e), which can also seen to be surrounding and innervating the VGlut2+ (red) glomeruli (f, i.e. arrows). Abbreviations as in Figure 1. Scale bars = 100 µm in a, c, e, and 25 µm in b, d, f.
(5.88 MB TIF)
