# Supplementary material for: Principles of Glomerular Organization in the Human Olfactory Bulb – Implications for Odor Processing
Source: PLoS One. 2008 Jul 9;3(7):e2640. doi: 10.1371/journal.pone.0002640 (PMC2440537; doi:10.1371/journal.pone.0002640)
Supplement: Table S1 — Olfactory bulb analysis data (0.05 MB DOC) [file pone.0002640.s001.doc]

**Supplementary Table 1**

|  |  | |  | |  | |  |  |
| --- | --- | --- | --- | --- | --- | --- | --- | --- |
| **Table S1: Olfactory Bulb Analysis Data** | | | | | | |  |  |
|  | |  | |  | |  |  |  |
| **ID** | | **OB Length (mm)** | | **Average Area of Slice (mm^2^)** | | **Total OB Volume (mm^3^)** | **Average Glomerular Diameter (µm)** | **Total Glomeruli (corrected)** |
| HOB 1 | | 7.05 | | 4.30 | | 30.32 | 65.13 | 4595 |
| HOB 2 | | 9.60 | | 5.99 | | 57.50 | 55.15 | 4184 |
| HOB 6 | | 9.50 | | 5.73 | | 54.44 | 56.17 | 2975 |
| HOB 7 | | 11.35 | | 4.39 | | 49.83 | 59.29 | 6530 |
| HOB 15 | | 9.40 | | 6.28 | | 59.03 | 57.44 | 7150 |
| HOB 16 | | 9.35 | | 5.64 | | 52.73 | 62.68 | 4221 |
| HOB 20 | | 10.40 | | 6.28 | | 65.31 | 61.37 | 9325 |
| **Mean ± S.E.M.** | | **9.52 ± 0.49** | | **5.52 ± 0.32** | | **52.74 ± 4.19** | **59.60 ± 1.41** | **5568 ± 830** |
|  | |  | |  | |  |  |  |
