# Supplementary material for: Principles of Glomerular Organization in the Human Olfactory Bulb – Implications for Odor Processing
Source: PLoS One. 2008 Jul 9;3(7):e2640. doi: 10.1371/journal.pone.0002640 (PMC2440537; doi:10.1371/journal.pone.0002640)
Supplement: Table S2 — Olfactory bulb donor information (0.04 MB DOC) [file pone.0002640.s002.doc]

**Supplementary Table 2**

| **Table S2: Olfactory Bulb Donor Information** | | | |  |
| --- | --- | --- | --- | --- |
|  |  |  |  |  |
| **OB Identification** | **Source Type** | **Age of Donor** | **Gender** | **Relevant Clinical Information** |
| HOB 1 | Surgery | 39 | F | frontal lobe epilepsy |
| HOB 2 | Post Mortem | 89 | M | lung adenocardinoma |
| HOB 6 | Post Mortem | 67 | M | leukemia treated with chemotherapy |
| HOB 7 | Surgery | 66 | M | pituitary tumor |
| HOB 15 | Post Mortem | 70 | M | emphysema |
| HOB 16 | Post Mortem | 85 | F | microscopic polyangiitis |
| HOB 20 | Surgery | 49 | F | frontal lobe glioma |
