# Supplementary figures and images for: Principles of Glomerular Organization in the Human Olfactory Bulb – Implications for Odor Processing
Source: PLoS One. 2008 Jul 9;3(7):e2640. doi: 10.1371/journal.pone.0002640 (PMC2440537; doi:10.1371/journal.pone.0002640)

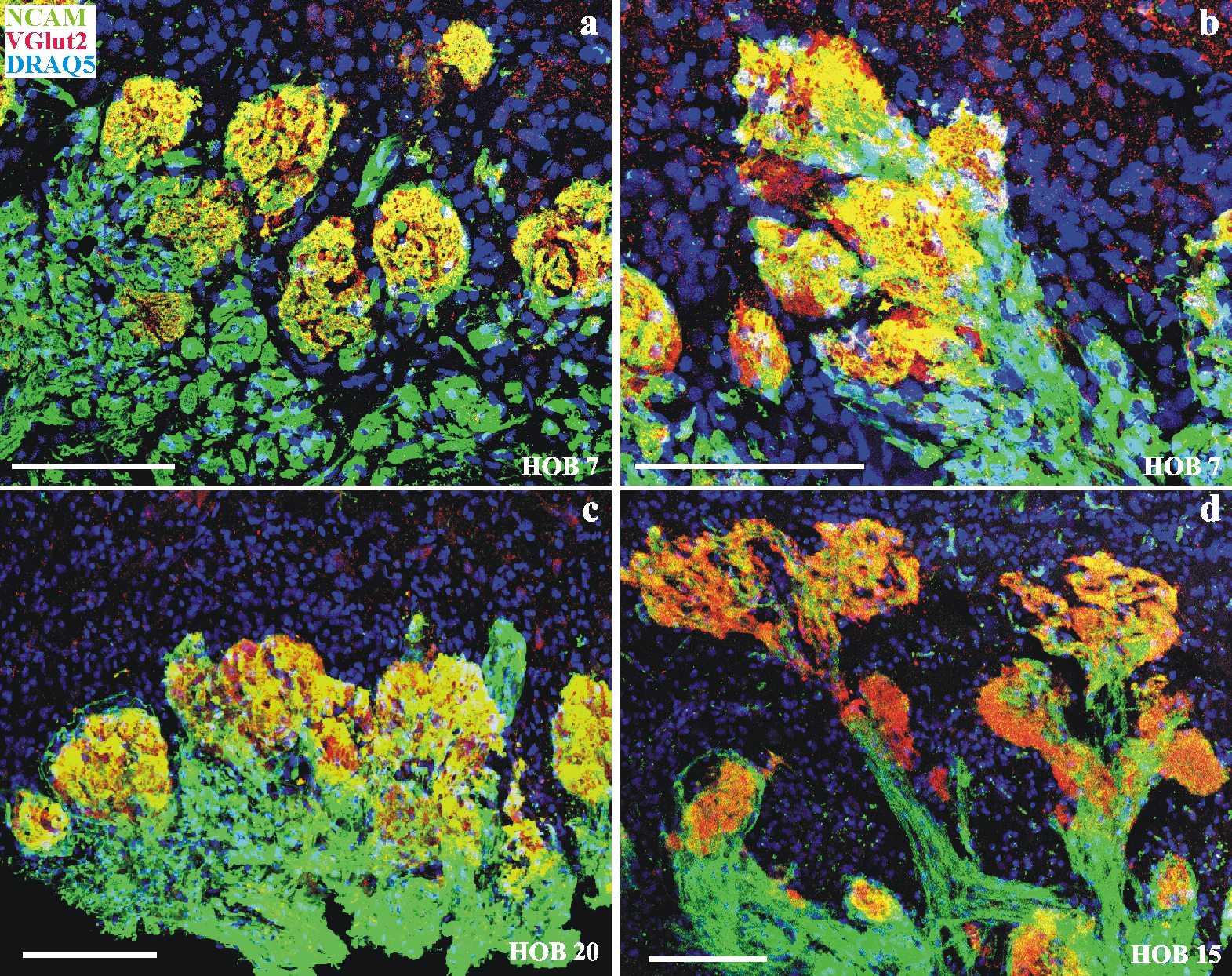

Supplement: Figure S1 — Additional glomerular phenotypes Additional examples of glomeruli from HOBs labeled with NCAM (green) and VGlut2 (red). Glomeruli were often regularly spherical and regularly distributed (a), though sometimes clustered in groups that make increase the difficulty of distinguishing individual glomeruli and their size and shape (b, c). A further example emphasizes the complexity of glomerular organization and penetration into the deep layers of the HOB (d). Scale bars are 100 µm in a–d. (5.87 MB TIF) [file pone.0002640.s003.tif]

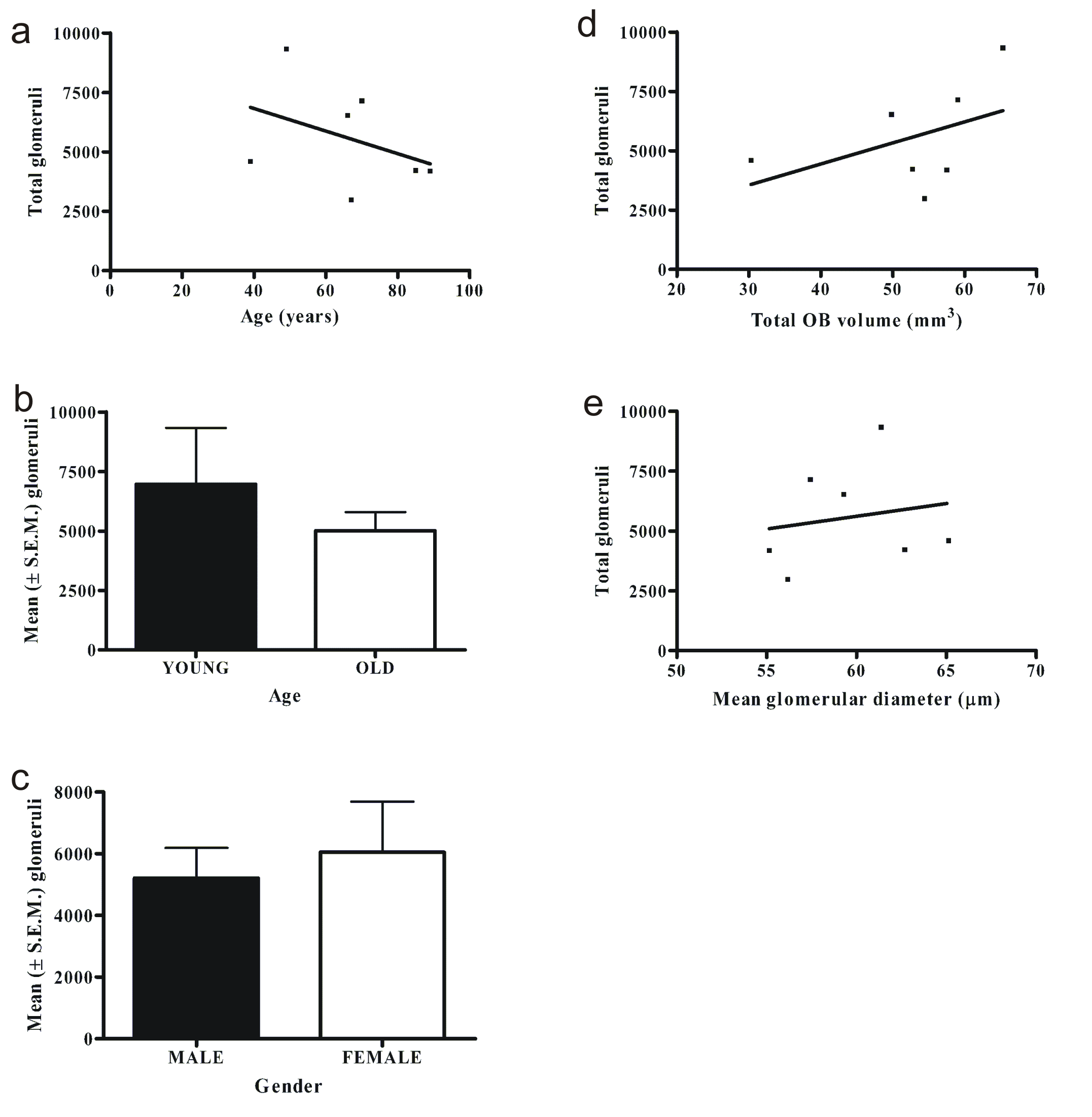

Supplement: Figure S2 — Relationships between total glomerular and age, gender, glomerular diameter, and OB volume No significant relationships were found between total glomeruli and donor age (p = 0.39) (a). There was a trend towards decreasing numbers of glomeruli with increasing age, however even when dichotomized into two groups of young (age less than 50 years old) and elderly (age greater than 50 years old), there was not a significant difference (p = 0.33). The average number of glomeruli in OBs from the young group was 6,960±2,365 (n = 2), while in the elderly group it was 5,012±785 (n = 5) (b). When grouped by gender, the mean number of glomeruli in OBs from female donors was 6,047±1,643 (n = 3), and from male donors, 5,210±981 (n = 4), which was also not significant (p = 0.66) (c). Finally, there was no correlation between glomerular number and average glomerular size (p = 0.71) (d), or between glomerular number and OB volume (p = 0.31) (e). Linear regressions were performed to look for significance in a, d, e. Unpaired t-tests were performed for the two-group comparisons in b, c. There were no significant differences in variance for either of these comparisons. (14.23 MB TIF) [file pone.0002640.s004.tif]

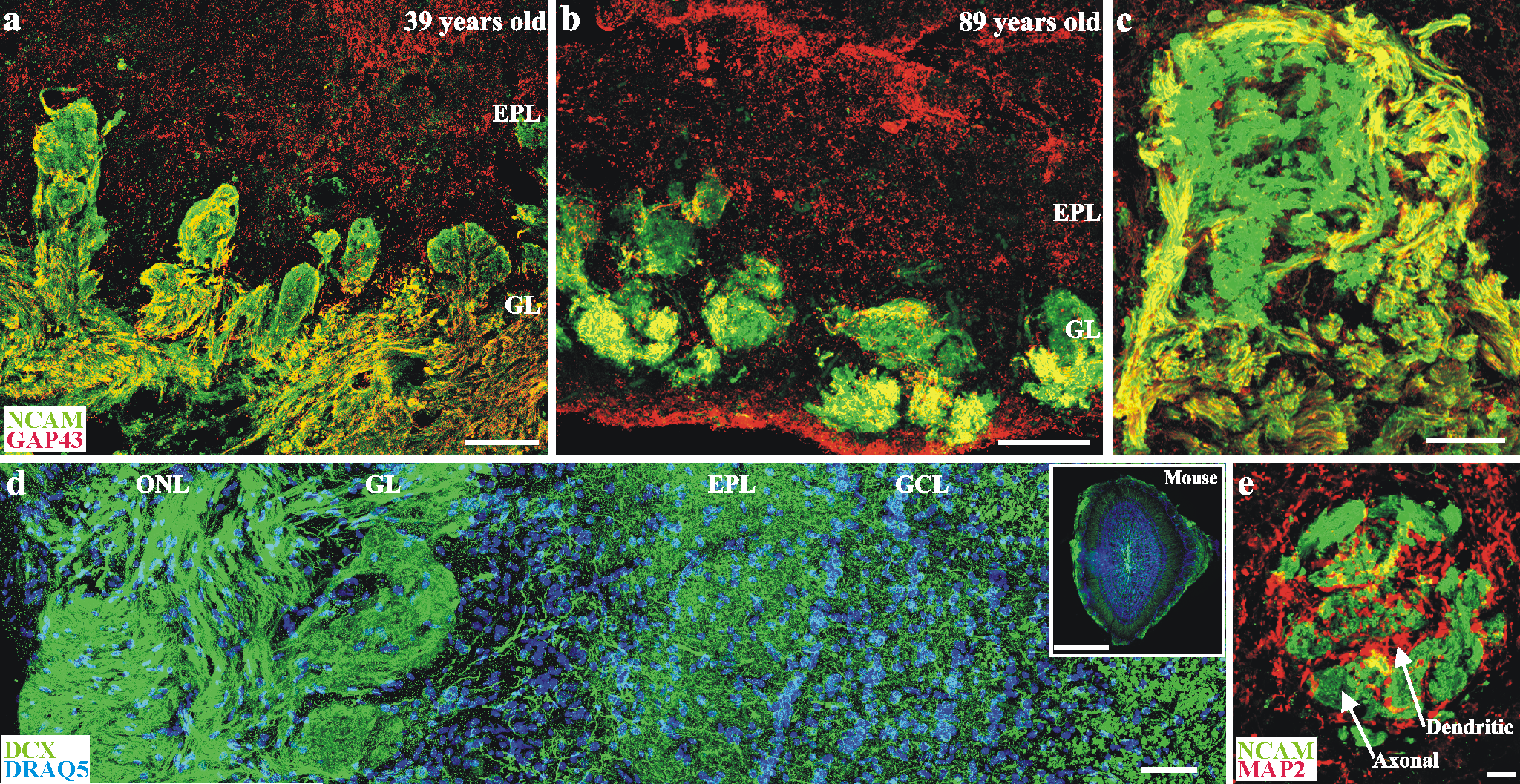

Supplement: Figure S3 — Neurogenesis and intrinsic organization of HOB glomeruli Double labeling with GAP43 (red) and NCAM (green) identifies immature OSN axons in the olfactory nerve layer and in the glomeruli of the HOB from both young (a) and older (b) donors. The immature GAP43+ OSN axons first integrate into the periphery of existing glomeruli, a process previously described in rodents (c). Immature OSN axons are also seen with doublecortin in both the nerve layer and glomeruli (d). Migrating neuroblasts, also identified with doublecortin (green), are seen in the human OB (d) as previously described in mice (inset). The presence of subcompartmental organization within glomeruli, axonal compartments as demonstrated by NCAM (green) and dendritic compartments as demonstrated by MAP2 (red) (e), suggests a further parallel with the intrinsic organization of glomeruli in rodents. Abbreviations as in Figure 1. Scale bars: a = 100 µm in a, b; 25 µm in c, e; 50 µm in d; 500 µm in inset of d. (6.50 MB TIF) [file pone.0002640.s005.tif]

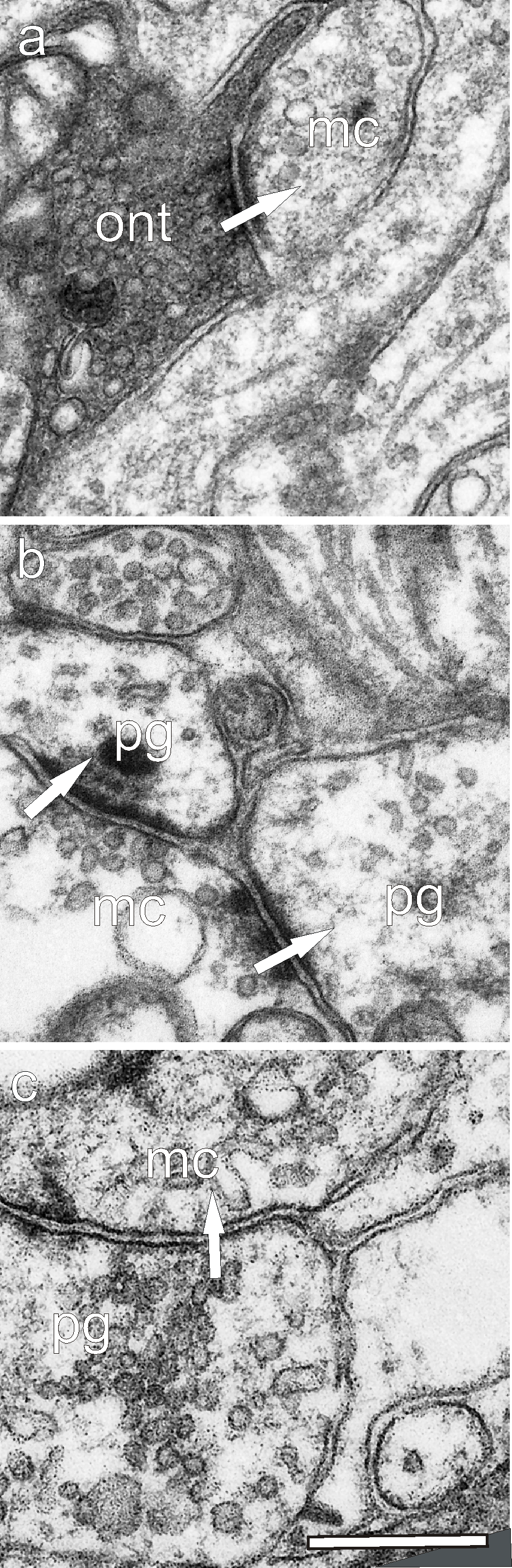

Supplement: Figure S4 — Synaptic morphology in HOB glomeruli (a) In the HOB olfactory nerve terminals (ont) make typical asymmetrical axodendritic synapses with OB neurons. Clusters of spherical vesicles are seen closely apposed to the presynaptic membrane in the electron dense axon terminals. (b) Mitral cell dendrites in the glomeruli make asymmetrical synapses with the intraglomerular dendrites of periglomerular cells. The clusters of vesicles in the mitral cell dendrite a characteristically small. (c) Periglomerular cell dendrites establish symmetrical synapses with mitral cell dendrites. Note the pleomorphic nature of the synaptic vesicles in the periglomerular cell dendrite. Arrows indicate the polarity of the synapses. Abbreviations: ont, olfactory nerve terminal; mc, mitral cell dendrite; pg, periglomerular cell dendrite. Calibration bar shown in (c) = 1 µm. (7.71 MB TIF) [file pone.0002640.s006.tif]

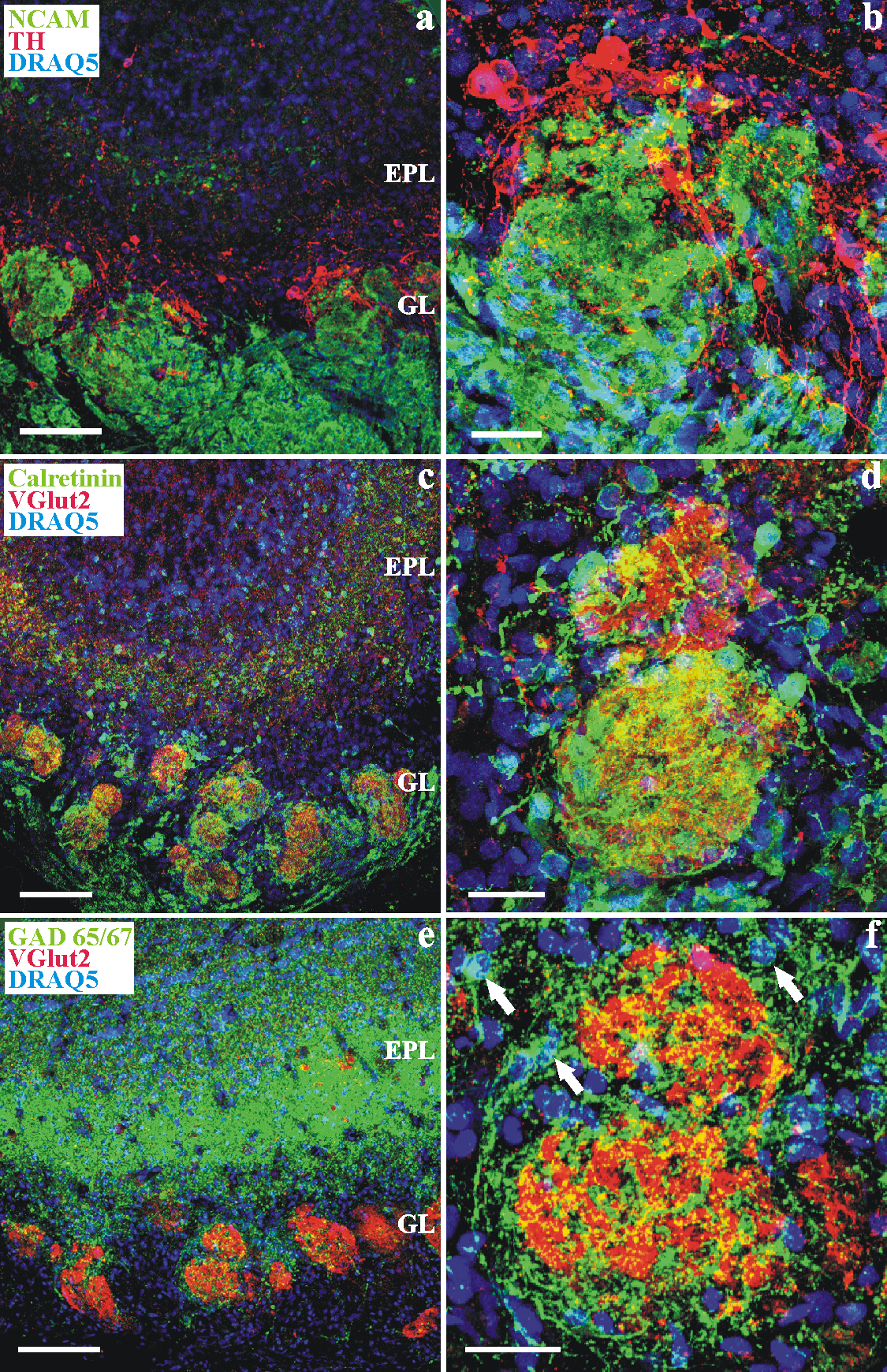

Supplement: Figure S5 — Molecular phenotypes and distributions of periglomerular cells In the HOB large TH+ (red) cells surround glomeruli, as defined by NCAM (green) and DRAQ5 (blue) (a) At higher magnification processes from the TH+ neurons are seen extending into a glomerulus (b). Calretinin+ (green) cells have smaller cell bodies and are densely distributed around the circumference of HOB glomeruli identified with VGlut2 (red) (c,d). The EPL is dense with GAD65/67+ (green) processes (e), which can also seen to be surrounding and innervating the VGlut2+ (red) glomeruli (f, i.e. arrows). Abbreviations as in Figure 1. Scale bars = 100 µm in a, c, e, and 25 µm in b, d, f. (5.88 MB TIF) [file pone.0002640.s007.tif]
